# Biomonitoring of inorganic arsenic species in pregnancy

**DOI:** 10.1038/s41370-022-00457-2

**Published:** 2022-08-10

**Authors:** Jillian Ashley-Martin, Mandy Fisher, Patrick Belanger, Ciprian Mihai Cirtiu, Tye E. Arbuckle

**Affiliations:** 1 Environmental Health, Science and Research Bureau, Health Canada, Ottawa, ON Canada; 2 INSPQ, Centre de toxicologie du Québec, Direction de la santé environnementale, au travail et de la toxicology, Quebec, QC Canada

**Keywords:** Arsenic, Biomonitoring, Cohort studies, Pregnancy, Speciated arsenic

## Abstract

Exposure assessment of inorganic arsenic is challenging due to the existence of multiple species, complexity of arsenic metabolism, and variety of exposure sources. Exposure assessment of arsenic during pregnancy is further complicated by the physiological changes that occur to support fetal growth. Given the well-established toxicity of inorganic arsenic at high concentrations, continued research into the potential health effects of low-level exposure on maternal and fetal health is necessary. Our objectives were to review the value of and challenges inherent in measuring inorganic arsenic species in pregnancy and highlight related research priorities. We discussed how the physiological changes of pregnancy influence arsenic metabolism and necessitate the need for pregnancy-specific data. We reviewed the biomonitoring challenges according to common and novel biological matrices and discussed how each matrix differs according to half-life, bioavailability, availability of laboratory methods, and interpretation within pregnancy. Exposure assessment in both established and novel matrices that accounts for the physiological changes of pregnancy and complexity of speciation is a research priority. Standardization of laboratory method for novel matrices will help address these data gaps. Research is particularly lacking in contemporary populations of pregnant women without naturally elevated arsenic drinking water concentrations (i.e. <10 µg/l).

## Introduction

Arsenic is a naturally occurring metalloid in soil and a leading global drinking water contaminant [1]. Inorganic arsenic (iAs) is a known carcinogen and iAs exposure is associated with cardiovascular disease, developmental toxicity, neurotoxicity, and diabetes [2–6]. High concentrations of total arsenic in pregnancy have been consistently associated with adverse maternal-child health outcomes including infant mortality [7], low birth weight [7, 8], gestational diabetes (GDM) [9], preterm birth and preeclampsia [10]. Nevertheless, the etiologic role of low-level total or iAs arsenic exposure in maternal-child health remains unclear. A primary challenge in this body of literature is exposure measurement; the complexity of arsenic metabolism combined with the physiological changes of pregnancy necessitates careful consideration of the appropriate analytes, biological matrices, and timing of measurement and interpretation of results.

Total arsenic is composed of both inorganic and organic species [11] (Table 1). iAs is commonly found in drinking water and many foods (e.g., rice, non-rice grains, vegetables, fruits, meats, dairy, seaweed) [12, 13] and largely consists of arsenate (pentavalent arsenic (As^V^)) and, to a lesser extent, arsenite (trivalent arsenic (As^III^)). These are the two most common valence states to which humans are exposed [14]. Once ingested, arsenic is metabolized via oxidative methylation, glutathione conjugation [15, 16] or via a more recently identified reductive methylation pathway [17]. Metabolism of the parent iAs species produces mono-methylated (monomethylarsonic acid (MMA^V^) and monomethylarsonous acid (MMA^III^)) and dimethylated (dimethylarsinite (DMA^III^) and dimethylarsinic acid (DMA^V^)) metabolites [15]. MMA^III^ and DMA^III^ are both highly unstable metabolites and more toxic than the pentavalent forms [2, 15, 18, 19]. In addition to being an end product of metabolism, DMA^V^ concentrations may also originate from directly consumed dimethylated arsenic (DMA) or metabolized organic arsenicals such as arsenolipids and arsenosugars [20]. Organic arsenic is commonly found in seafood and includes arsenosugars, arsenolipids, and arsenobetaine [18]. Although organic arsenic is relatively nontoxic, it is possible that certain organic arsenicals may demethylate into more toxic parent compounds [21, 22]. When water iAs concentrations are elevated (e.g., >10 ug/l) such as in endemic regions of arsenic poisoning, exposure to iAs from drinking and cooking water exceeds exposure via food. In this high iAs exposure scenario, multiple arsenic biomarkers including iAs, iAs methylation metabolites (MMA, DMA), and total As all may be considered to be reliable measures of oral iAs exposure. In contrast, when water iAs concentrations are lower (<10 µg/l), iAs exposure from food and water is comparable to exposure levels from dietary sources of organic arsenic (described above, Table 1) resulting in challenging interpretation of arsenic biomarkers [23].

**Table 1:** Common sources of arsenic species.

	Species	Exogenous sources	Endogenous production	Notes
Inorganic arsenic				
Parent compounds	As^V^	Drinking water, multiple foods in diet		Known carcinogen
	As^III^	Drinking water, multiple foods in diet	Reduction of As^V^	Known carcinogen
Methylated metabolites	MMA^V^	Directly consumed DMA in foods	Methylation of As^III^	
	MMA^III^		Reduction of MMA^V^	More unstable and toxic than MMA^V^
	DMA^III^		Methylation of MMA^III^	More unstable and toxic than DMA^V^
	DMA^V^		Oxidation of DMA^III^ Demethylation of organic arsenicals	Primary metabolite in urine
Organic arsenic	Arsenosugars Arsenolipids Arsenobetaine Arsenocholine	Seaweed, fish, mollusks, crustaceans, cephalopods		Organic arsenicals are relatively nontoxic
Total Arsenic = ∑ Inorganic + organic + methylated metabolites				

*As*^*V*^ arsenate, *As*^*III*^ arsenite, *MMA*^*V*^ monomethylarsonic acid, *MMA*^*III*^ monomethylarsonous acid, *DMA*^*III*^ dimethylarsinite, *DMA*^*V*^ dimethylarsinic acid, *DMA* dimethylated arsenic.

Studies that measure arsenic concentrations in pregnancy face particular methodological challenges [24–30]. Normal physiological changes of pregnancy such as increased glomerular filtration rate (GFR) [25, 26] and plasma volume expansion [27, 28] complicate interpretation of biomonitoring data in urine and blood as does the presence of pregnancy complications (e.g., GDM, preeclampsia) [29]. Biomonitoring during pregnancy, however, informs risk assessments and is fundamental to epidemiological studies of maternal and child health. Compared to other life stages, pregnant women and children are more vulnerable to the adverse effects of environmental chemicals, including arsenic [31–33]. Although the broad challenges of biomonitoring in pregnancy have been previously elucidated [30, 34], arsenic exposure assessment warrants particular focus.

Our objectives were to review the value of and challenges inherent in measuring iAs species in pregnancy. We conclude with a discussion of data gaps and research priorities. We focus on iAs species due to their known toxicity. Previous reviews of arsenic biomonitoring focused on exposure in relation to drinking water [14] and within a highly exposed community in Mongolia [35]. The one identified publication related to exposure in pregnancy focused on maternal-fetal transfer of arsenic in a highly exposed Bangladeshi population [36].

## Value of measuring arsenic species in pregnancy

According to biomonitoring studies in the North America, Europe, and Asia, the vast majority of pregnant women have detectable concentrations of total arsenic in blood and urine [37–44]. Total arsenic is, however, prone to misclassification and misinterpretation because it is a sum of multiple species with differing toxicity profiles [45]. For example, the cytotoxicity of pentavalent and trivalent iAs species and their metabolites varies by up to five orders of magnitude [46]. Arsenobetaine, which has low toxicity [18], may represent a significant portion of total arsenic in urine and bias interpretation of biomonitoring data. Thus, arsenic toxicity is determined by both the dose of exposure as well as the relative concentrations of inorganic and organic arsenic species and metabolites. Compared to estimates of total arsenic, data on speciated arsenic in pregnancy are sparse yet necessary to capture the unique changes in metabolism, exposure profiles, and corresponding health risks.

The efficiency of arsenic metabolism and methylation increases throughout pregnancy [47–50] partly due to rising estrogen levels that upregulate choline [51]. Choline is needed to meet fetal nutrient needs for tissue growth and brain development [52] and is metabolized to betaine which can act as a methyl donor [53, 54]. Heightened methylation tends to increase the relative percentage of DMA compared to other species in urine [47]. For example, the mean percent of DMA in urine is 60–80% [2, 55, 56] in non-pregnant populations and 71.0–89.7% in pregnant populations [49, 54, 57, 58] with urine samples collected in first (<12 weeks) [49, 54], second (<27 weeks) [49, 54], third trimester [59] and delivery [57, 58]. In contrast, the mean percent of urinary MMA in non-pregnant adults is 10–20% vs. 5–11% in pregnant women [54, 57, 58, 60]. These data are largely from highly exposed populations in Bangladesh and may not be generalizable to populations with lower exposure levels. Moreover, the potential hormonally induced changes in arsenic methylation are one of many factors that may influence speciation profile during pregnancy. For example, in non-pregnant populations, adiposity as measured by body mass index (BMI), has been positively associated with %DMA and inversely associated with %MMA [61, 62]. These associations may be explained by the impact of adipose tissue on estrogen levels, subsequent choline production and resulting heightened arsenic methylation efficiency [62]. Despite these findings, similar associations between adiposity and arsenic speciation profile have not been observed during pregnancy [49, 54]. We hypothesize that the influence of pregnancy-induced changes in estrogen levels—and corresponding choline production—surpasses the potential corresponding changes induced by adipose tissue. To our knowledge, the previously mentioned pregnancy-related changes in kidney function do not induce changes in speciation profile.

The association between arsenic and pregnancy complications is likely dependent upon a woman’s speciation profile [63]. Authors of a review of arsenic, cancer and cardiometabolic health concluded that individuals with higher DMA relative to MMA experienced an increased risk of type 2 diabetes whereas women with the opposite profile (higher MMA relative to DMA) had increased risk of cancer and cardiovascular disease [64]. This finding supports the hypothesis that higher DMA relative to MMA during pregnancy may correspond with an increased risk of gestational diabetes; however, data are lacking. In the one identified study of methylation capacity and pregnancy outcomes, authors of a recent case-control study reported that women with higher %DMA had a decreased risk of GDM but all estimates were imprecise and included the null value [65].

## Measurement of inorganic arsenic species: methods and challenges

The half-life, bioavailability, and ability to measure iAs species varies according to biological matrix (Table 2). We review the challenges of measuring iAs in the following biological matrices: urine, blood, nails and hair, umbilical cord blood, placenta, meconium, saliva, teeth and bone, and human milk.

**Table 2:** Biological matrices used for human biomonitoring of arsenic during pregnancy.

Matrix	Pros	Cons
Urine	• Established methodology for measuring iAs species • Non-invasive	• Short half-life (days) • Measure of excretion and recent exposure • Requires correction for hydration • Low detection rates for iAs species
Blood	• Represents exogenous exposures and tissue burden • Steady-state concentration among women with chronic, high exposure • High detection rates • Indicator of ingested dose	• Short half-life (hours) • Represents short term exposure in low-exposed populations • Not an indicator of chronic exposure in low-exposed populations • Invasive • Provides an indication of total As • Limited methods for measuring specific species
Toenails	• Indicator of chronic iAs exposure • Non-invasive • Easy to ship and store • Long half-life • Established methodology for measuring total iAs and some species	• Distal to target organ • Questionable biological relevance • Lack of reference material • Lack of standard processing protocol • Subject to external contamination
Meconium	• Possible indicator of exposure throughout mid to late pregnancy • Non-invasive	• Questionable relevance to maternal exposure • Variability in analytic methods • Meconium matrix is variable among samples • No identified speciation measurements • Lack of reference material
Hair	• Indicator of chronic iAs exposure • Facilitates estimates of exposures in recent months • Non-invasive • Easy to ship and store • Established methodology for measurement iAs and some species	• Distal to target organ • Questionable biological relevance • Subject to external contamination
Placenta	• Non-invasive • Available at delivery • Can measure biomarker of effect (DNA methylation) in same tissue	• Low correlations compared to nail and urine concentrations • Measurement of total not speciated arsenic • Concentrations vary across tissue
Cord blood	• May reflect in utero exposure for neonates • Available at delivery	• Not reflective of maternal exposure • Large interindividual variation in maternal-fetal transfer • Measurement of total not speciated arsenic • May be difficult to collect
Saliva	• Non-invasive • Collection can be done in non-clinical setting • Easy storage • Established methodology for measurement iAs and some species • Moderate to strong correlations with total urine and toenail As in highly exposed populations	• Hasn’t been performed in pregnant population • Questionable value in low-exposed populations • Different speciation profile than in urine • Low concentrations and variability between individuals

### Urine

Urine is the most well-established matrix for measuring speciated iAs [44, 66, 67]. Despite its ease of collection, urinary measurements have a relatively short-half life (4 days), reflect recent exposure, are considered more a measure of excretion than body burden, and require correction for hydration status [66, 67]. Variability in urinary arsenic concentrations across pregnancy tends to be higher than variability in non-pregnant populations. Authors of a study that measured total urinary arsenic in all three trimesters reported an intraclass correlation coefficient (ICC) of 0.12 suggesting high variability across pregnancy [68]. In studies of non-pregnant adults, ICCs for repeated urinary arsenic measures ranged from 0.35 to 0.49 for inorganic species [69, 70]. Urinary biomonitoring data are also potentially influenced by the time of collection, preservation methods, and transportation [71].

Urinary arsenic tends to be dominated by DMA. In pregnancy cohort studies from Bangladesh [47, 49, 54, 57], Spain [72], China [73], Mexico [58] and Chile [48], the average %DMA ranged from 71.0–89.7 whereas %MMA ranged from 4.8–7.8. The percent of inorganic species ranged from 4.7 to 20.6 [48, 73]. In the one study that examined As^III^ and As^V^ separately, Gao et al. [47] reported 0% As^III^ and 2.1–2.6% As^V^. In studies with repeated measurements, %DMA increased by gestational age (GA) in pregnant women from Chile (mean GA 19.6: %DMA = 80.9; mean GA 35.7: %DMA = 86.3) [48] and Bangladesh (mean GA 8: %DMA = 73; mean GA 30 %DMA = 83) [49] but not in the study from China (mean GA 12.9 %DMA = 72.2; mean GA 34.2 %DMA = 71.0) [73]. The high percent of DMA in urinary arsenic creates challenges to using the sum of iAs, MMA, and DMA as a biomarker of iAs exposure in populations with moderate or high seafood consumption. As previously noted, DMA may originate from metabolized organic arsenicals that are found in seafood [20, 74]. Researchers that aim to estimate iAs exposure, therefore, need to account for seafood intake by statistically adjusting for DMA, arsenobetaine, or fish intake in regression models, calibrating biomarkers by correcting for model residuals, or restricting analyses to individuals who did not eat fish [74, 75]

There is considerable variability in detection rates and limits of detection (LOD) among studies that measured speciated iAs in pregnancy (Table 3). Detection rates for urinary concentrations of parent compounds (As^V^ and As^III^) and MMA are typically low in populations without high exposure levels [38, 76]. Detection of As^III^ varied from 10.9–87.4% in identified studies of iAs speciation in pregnancy (Table 3). Trivalent metabolite species (MMA^III^, DMA^III^) are rarely detected as these species are unstable and easily convert depending on the analytical pH (As^III^ vs. As^V^). In some cases, chromatographic peaks related to DMA^III^ and MMA^III^ can be identified but new chromatographic methods and corresponding reference standards are needed to facilitate routine detection of these unstable species.

**Table 3:** Lab and specimen details in studies that measured urinary speciated arsenic in pregnancy.

					Detection frequencies (% >LOD)				
Reference	Sample size	Methods	Urine collection period^a^	LODs	Arsenate (As^V^)	Arsenite (As^III^)	MMA	DMA	AsB
Bangladesh cohort study (Gao et al. [47])	1613	HPLC and hydride generator atomic absorption spectrometry	Visit 1: 4–16 Visit 2: 21–37	AsIII 0.02, AsV 0.06, MMA 0.07, DMA 0.10 (µg/l)	Visit 1: 50 Visit 2: 42	Visit 1: 70 Visit 2: 74	Visit 1: 80 Visit 2: 88	Visit 1: 100 Visit 2: 100	NR
BEAR study (Laine et al. [58])	197	HG-CT-AAS	Delivery	iAs 0.2, MMA, 0.1, DMA 0.1 (µg As/l)	NR	NR	97	100	
China cohort study (Wang et al. [73])	1038	HPLC coupled to ICP-MS	Visit 1: 12.9 (1.0)^b^ Visit 2: 23.9 (3.6) Visit 3: 34.2 (3.3)	AsIII 0.3, AsV 0.3, MMA 0.1, DMA, 0.1, AsB 0.1 (µg/l)	NR	NR	72.3	99.3	
China cross sectional study (Zhang et al. [136])	396	HPLC coupled to ICP-MS	24–28	AsIII 0.2, AsV 0.5, MMA 0.2, DMA 0.2, AsB 0.2 (µg/l)	99.2	87.4	9.3	99.7	99.2
INMA study (Soler-Blasco et al. [72])	1017	HPLC coupled to ICP-MS	13 (1.2)	iAs 0.02–0.03, MMA 0.03, DMA 0.03, AsB 0.02 (µg/l)	NR	NR	97.2	100	100
Los Angeles cohort study (Howe et al. [75])	167	HPLC coupled to ICP-MS	14 (4)	AsIII 0.011–0.04, AsV 0.02–0.143, MMA 0.020–0.086, DMA 0.014–0.169 (µg/l)	58.1	49.7	77.2	98.8	71.3
MIREC study (Ettinger et al. [38])	1933	HPLC coupled with ICP-MS	6.1–14.9	0.75 µg As/l	1.5	15.9	7.5	85.9	48.8
National Children’s Study (Shih et al. [76])	112–212^c^	HPLC coupled to ICP-DRC-MS	Third trimester	AsB 0.4, DMA 1.7, AsIII 1.2, AsV 1.0 (µg/l)	<40	<40	<40	76.9	<40
New Hampshire birth cohort (Farzan et al. [100])	1151	HPLC coupled to ICP-MS	24–28	0.10–0.15 µg/l	NR	NR	83.2	99.5	NR
Oklahoma case-control study (Chen et al. [65])	64 cases 237 controls	HPLC coupled to ICP-DRC-MS	24–28	AsIII 1.25, AsV 0.48, MMA 0.87, DMA 0.89, AsB 1.80 (µg/l)	Cases 18.8 Controls 15.6	Cases 10.9 Controls 14.8	Cases 9.4 Controls 11.0	Cases 68.8 Controls 76.4	Cases 25.0 Controls 23.2
Peru birth cohort (Fano-Sizgorich et al. [137])	147	ICP-MS	15.7(4.7)	0.1 (µg/l)	100	100			
Taiwan birth cohort (Chou et al. [138])	299	HPLC-ICP-MS	28–38	0.09 AsIII, 0.05 AsV, 0.05 MMA, 0.04 DMA (µg/l)	93.3	95.3	96.6	96.0	NR

*AsB* arsenobetaine, *DRC* dynamic reaction cell, *HG-CT-AAS* hydride generation cryotrapping atomic absorption spectrometry, *HPLC* high performance liquid chromatography, *iAs* inorganic arsenic, *ICP-MS* inductively coupled mass spectrometry, *INMA* INfancia y Medio Ambiente, *MIREC* Maternal-Infant Research on Environmental Chemicals, *NR* not reported, *MMA* monomethylarsonic acid, *DMA* dimethylated arsenic.

^a^Time of collection is presented as weeks gestation unless otherwise noted and is reported as either a range or mean (SD) depending on the reporting style in the publication.

^b^One detection rate was provided for all three visits.

^c^Number varied depending on outcome.

Most studies that measured iAs have used high performance liquid chromatography (HPLC) inductively coupled plasma with mass spectrometry (ICP-MS). Chemists use HPLC for chromatographic separation of each species and ICP-MS for detection. Even among studies that use this approach, it is not possible to compare sensitivities or the impact of potential interferences, in the absence of detailed lab-specific procedures. Laboratories have different approaches for determining and reporting the LOD and different chromatographic conditions. Furthermore, although labs may produce results with low LOD (sub parts per billion), this sensitivity is not necessarily persistent over time. The overall advantages of the HPLC-ICP-MS approach are good reproducibility over time, good specificity and sensitivity (signal to background noise), and identification of a profile of species. The method is also useful for comparing biomonitoring results within the same study or with other studies. The analytic costs of measuring speciated arsenic are higher than total arsenic.

**Fig. 1:**
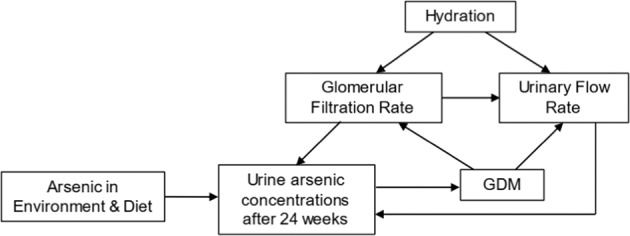
Conceptual model illustrating estimation of urinary arsenic concentrations and risk of GDM. Estimating associations between urinary arsenic concentrations and GDM may be biased by insufficient adjustment for urinary hydration; dilute urinary samples will underestimate concentrations whereas concentrated samples will overestimate exposure. This potential bias is particularly problematic when sampling occurs subsequent to the onset of physiological changes associated with GDM such as increased urinary output and glomerular filtration rate. In this figure, we have indicated this onset as after 24 weeks as this is typically the earliest time of GDM diagnosis.

One of the key challenges of measuring urinary arsenic, particularly during pregnancy, is exposure misclassification due to heterogeneity in urinary dilution [77]. Pregnancy has profound impacts on kidney function. In healthy pregnancies, GFR increases 50%, kidney length increases by 1 to 1.5 cm, kidney volume increases 30%, and hydronephrosis occurs in nearly half of women [24]. Pregnancy complications exacerbate the challenges associated with kidney function and urinary dilution. Compared to women without disruptions in glucose homeostasis, those with GDM, for example, tend to have higher GFR and urinary output, resulting in more dilute urine and artificially lowered urinary arsenic concentrations (Fig. 1). Correction for urinary dilution is critical to ensure appropriate comparison of urine concentrations between women [77]. Inadequate control for urinary dilution may lead to inaccurate estimates of the association between urinary arsenic concentrations and risk of GDM particularly when urine is collected at the time of or subsequent to GDM diagnosis. Standardization and/or adjustment for creatinine and specific gravity are both commonly used methods [78–80] to account for urinary dilution; however, specific gravity based techniques are preferable in pregnancy because creatinine levels are influenced by pregnancy and pregnancy complications [81]. Furthermore, specific gravity has been shown to have higher within-person reproducibility and lower systemic variation than creatinine among pregnant women [82]. In a non-pregnant population, creatinine and specific adjusted total urinary arsenic were both positively correlated with total blood arsenic concentrations (*p* for both correlation coefficients <0.001); the correlation with the creatinine adjusted concentrations was moderately higher. The authors concluded that creatinine adjusted concentrations were a better predictor of blood arsenic concentrations but this conclusion may not be applicable to pregnant populations [83]. Interindividual differences in urinary flow rate can also influence biomarker concentrations [84]. Collection of these data (total volume voided and time since last void) and correction for flow rate may help overcome potential resulting biases in exposure assessment that are not addressed by creatinine or specific gravity based corrections because these methods cannot account for interindividual differences in flow rate. For example, in an analysis of NHANES data, Hays et al. [85] demonstrated that analyses of chemical concentrations and BMI may be subject to reverse causality when body weight adjusted flow rate is not considered. Christenen et al. [86] have addressed this potential bias by using chemical excretion rate, calculated as the product of the urine flow rate and the metabolite concentration, as the exposure variable of interest.

### Blood

Given the frequency with which women are in contact with clinical care during pregnancy, blood collection is often feasible [67, 87] and detection rates of total arsenic in studies of pregnant women have been relatively high (>90%) [88–90]. Blood arsenic is influenced by tissue compartment concentrations and exogenous exposure and reflects overall body burden and ingested dose [14, 67]. In populations without chronic arsenic exposure, blood concentrations reflect recent rather than chronic exposure because arsenic is cleared from blood several hours after absorption [14, 66, 67]. In populations with continuous arsenic exposure in drinking water [67, 87], blood concentrations will reach a steady-state and are indicative of chronic exposure levels [67].

Speciation in blood has been performed in a limited number of studies [91–93]. This analysis may not be feasible in populations with low levels of arsenic exposure and may not provide complete speciation. Proteins present in blood can bind to arsenic species, introduce analytical artifacts and decrease the sensitivity of the methods. Gamble et al. [91] used HPLC coupled with ICP-MS with dynamic reaction cell (DRC) technology (ICP-MS-DRC) to measure arsenobetaine, arsenocholine, MMA, DMA, As^III^ and As^V^ in samples with total arsenic concentrations higher than 3 µg/l. In contrast to the concentrations observed in the Gamble et al. study in Bangladesh [91], the Canadian Maternal-Infant Research on Environmental Chemicals (MIREC) study reported 95th percentiles of total blood arsenic of 2.32 and 2.77 µg/l for first and third trimester, respectively [38]. The method used by Gamble et al. [91] to detect speciated iAs has not been applied to populations with the low exposure levels typical of contemporary North America. Matousek et al. used hydride generation with cryotrapping coupled to ICP-MS to measure speciated iAs in blood [92]. This method provided summary measures of the iAs species (iAs^III^ + iAs^V^), mono and dimethylated metabolites but cannot perform complete speciation and measure all species separately. In addition, the authors did not detect arsenobetaine [92]. The one identified study that assessed arsenic speciation in the blood of pregnant women reported that DMA was the dominant species; mean %DMA, %MMA, %AsIII, and %AsV were 43.5, 30.1, 13.0, and 20.9, respectively [57]. In two non-pregnant populations from Bangladesh, arsenic species were distributed almost evenly among iAs, MMA, and DMA. The average %iAs, %MMA, and %DMA ranged between 26.0–29.8%, 37.6–43.9%, and 28.9–34%, respectively [57, 62].

Blood arsenic concentrations may be influenced by the normal physiological changes of pregnancy. For example, as GFR increases throughout pregnancy, blood chemical concentrations may be lowered as more chemicals are excreted in urine. Associations between chemical concentrations and pregnancy outcomes may be confounded by GFR when both the exposure (e.g., blood chemical concentrations) and the outcome (e.g., birth weight) are influenced by GFR or factors (e.g., pregnancy complications) that impact GFR [25, 94]. The extent and direction of this bias is particularly challenging to understand and address with arsenic due to potential bidirectional effects between arsenic and renal function [95, 96]. Plasma volume expansion increases ~45% throughout pregnancy; the resulting hemodilution of serum proteins may artificially lower concentrations of protein-bound environmental chemicals [27, 28]. Arsenic can bind to proteins in the blood such as hemoglobin and transferrin [46]. Late pregnancy measurements may be underestimated because of this binding and hemodilution. In the MIREC study, for example, median total blood arsenic concentrations were 0.112 µg/l higher in the first than the third trimester [38]. There are insufficient data on serial blood iAs measurements throughout pregnancy to understand how hemodilution may influence concentrations.

Although whole blood is the most traditional method of measuring arsenic in blood, authors have explored plasma [83] and erythrocytes [97] as potential matrices in non-pregnant populations. Further research is necessary to understand the potential utility of these measures in pregnancy.

### Nails and hair

Arsenic binds to the keratin in both nails and hair. Toenails provide a reliable means of assessing chronic iAs exposure due to their slow growth and the long half-life of iAs toenail concentrations (12–18 months) [45, 98, 99]. iAs has been detected in the majority (90%) of maternal toenail concentrations in two pregnancy cohort studies [45, 100]. A small study of non-pregnant participants (*n* = 47) measured iAs species in fingernails using HPLC coupled with ICP-MS and reported that As^III^ was the dominant species (58.6%), followed by As^V^ (21.5%), DMA^III^ (9.2%), MMA^V^ (7.7%) and DMA^V^ (3.0%) [101]. These findings suggest that speciation in nails is possible even if iAs binds more prominently to keratin than the metabolites [101]. In another analysis of the same population, the authors noted that nails adsorb about 1–2% iAs^III^, and 1–1.5% iAs^V^ suggesting that arsenic speciation in nails may be more related to the total (As^III^, As^V^ plus metabolites) rather than the parent iAs (As^III^, As^V^) concentrations [101]. Analyses and interpretation of toenail concentrations are limited by the lack of standard reference material and processing protocols. Furthermore, it is difficult to identify the precise window of exposure captured by toenail concentrations. Toenails are estimated to grow 0.03–0.05 mm per day but interindividual differences in growth rate and clipping length contribute to variability in corresponding time windows [45, 102].

Although biologically similar to nails, hair samples may have differing speciation profiles and carry their own distinct challenges. The previously mentioned study of iAs species in nails reported similar distributions in hair (As^III^ 60.9%, As^V^ 33.2%, MMA^V^ 2.2%, DMA^V^ 3.6%) yet no detectable DMA^III^ [101]. Hair may be more reflective of iAs than metabolites as it has been shown to adsorb 9–13% As^III^, and 12–16% As^V^ [71]. Both toenail and hair concentrations are distal to the target organs of interest [88]. Hair analyses face the additional challenge of contamination due to exogenous exposure (i.e., via dust, hair products, washing in contaminated water) [14, 45, 103]. Hair sample analysis is also subject to inter- and intra-laboratory variability and lack of reproducibility [104]. Consistency regarding processing protocol, the sample length and hypothesized window of exposure also varies. Previous studies that measured iAs in hair used 0.3 cm as an indicator of exposure over the previous 2 months [105] as well as 5.0 cm as an indicator of past 5 months [106].

In contrast to blood and urine, a single nail or hair measurement may be a more suitable indicator of exposure throughout pregnancy. For example, a small (*n* = 52) study of Bangladeshi women reported a strong correlation (*r* = 0.73) between maternal hair concentrations measured in the first (range: 6.5–25 weeks) and second visit (2 weeks postpartum) and a moderate correlation (*r* = 0.49) between maternal nail concentrations at the same two time points [102]. Consistent with these findings, a larger study from Bangladesh (*n* = 1613) reported strong correlation (*r* = 0.84) between maternal nails collected between 4–16 weeks and those collected postpartum [47]. These two studies reflect high exposure environments and may not be generalizable to other environments. Studies examining the temporal stability of hair and nail concentrations in low-exposed populations are necessary to determine if one postpartum measurement reflects exposure throughout pregnancy. Moreover, although a biomarker that represents average gestational exposure is advantageous from logistical and financial perspectives, there are drawbacks to not capturing the fluctuations in speciation profiles and concentrations that would be evident from serial biomarker measurements.

### Placenta

Arsenic readily crosses the placenta yet studies using placenta as a matrix for measurement are scarce. Placental total arsenic concentrations have low to moderate correlations with more established matrices (*r* = 0.21 with blood [107]; *r* = 0.11 with urine [108]) and, therefore, questionable interpretation. The primary advantage of placental concentrations is the ability to investigate total arsenic exposure and potential toxicity (gene expression and DNA methylation) in the same organ [108]. For example, placental arsenic was associated with 163 DNA methylation sites in the placenta; urinary and toenail concentrations were associated with zero and one site, respectively [109].

The use of placental arsenic in studies of other maternal endpoints is questionable. For example, authors of the Environment and Child (INMA) Project reported that only 27% of placentas had detectable concentrations of arsenic and observed no associations with birth outcomes. These authors did not evaluate arsenic in other matrices [110]. No studies of speciation in placental tissue were identified.

The heterogenous nature of placental tissue poses another challenge. Placental tissue is a mixture of blood, vessels, chorionic villi and membranes of both maternal and fetal origin. Measurements of placental arsenic concentrations need to ensure that the biopsies are representative of concentrations throughout the tissue and, preferably, remove any maternal tissue prior to sampling [111, 112].

### Cord blood

The primary advantage of obtaining umbilical cord blood arsenic concentrations is the ability to investigate links with neonatal health outcomes and maternal-fetal transfer. Total arsenic concentrations tend to be lower in cord than maternal blood [38, 107, 113]. Studies of arsenic transfer have reported cord to maternal blood ratios of 0.70 to 0.88 [107, 113]. Correlations between cord blood and total As during pregnancy in the MIREC study were low (first trimester: cord blood *r* = 0.19; third trimester: cord blood *r* = 0.29) [38]. No studies of speciation in cord blood were identified, yet methods for measuring speciation in blood could feasibly be applied.

### Meconium

Commonly used to detect fetal exposure to drugs, meconium forms in the 13th week of gestation and concentrations within this matrix may indicate cumulative fetal exposure throughout pregnancy [114–116]. Meconium is a heterogeneous material and thorough sample mixing is advised to ensure that the measured concentration is reflective of the entire sample. Chemical concentrations may also differ depending on the timing of meconium collection. Although no identified studies have observed this variability in arsenic, Ortega Garcia et al. reported differing concentrations in polychlorinated biphenyls and organochlorine pesticides according to the timing of meconium collection [117]. It is also difficult to separate meconium from the diaper and minimize potential contamination due to urine [30, 115]. Analytic methods for sample collection, laboratory analysis and reporting are not standardized which impedes comparison of results across studies [114].

Meconium arsenic detection rates have varied from 7% in the MIREC study (LOD: 0.225–0.599 µg/l) [38] to 100% in a case-control study in Xiamen, China [118] (LOD: 0.06 µg/l). Furthermore, while arsenic meconium concentrations may have relevance to neonatal exposure, they are unlikely to reflect maternal exposure particularly during early pregnancy.

### Saliva

No identified study measured salivary arsenic in pregnant women, yet it has been explored as a biomarker for speciated iAs in both children and non-pregnant adults in highly exposed populations [119, 120]. Collecting saliva is non-invasive and could be performed in a non-clinical setting [119, 120]. Limited data suggest that the half-life of arsenic species in saliva is comparable to urine (72 h) [121]. Studies in non-pregnant populations have measured iAs using HPLC-ICP/MS and reported moderate to strong correlations with more established matrices. Correlations between salivary and urinary arsenic were 0.50 [120] and 0.79 [119]. However, authors of a study in a region of arsenicosis (Shanxi, China) reported that salivary concentrations were lower and exhibited a different speciation profile than that observed in urine [119]. Specifically, the salivary species had a lower percentage of DMA and MMA and a higher percentage of iAs than in urine. It is yet unclear whether saliva is a suitable matrix for assessing exposure in pregnancy particularly in populations without elevated exposure.

### Teeth and bone

Teeth and bone are not commonly used matrices for arsenic biomonitoring. One study of 43 children living near a smelter in California reported that only 50% of children had detectable concentrations of arsenic in shed deciduous teeth [122]. In a study of organ deposition of inhaled arsenic using physiologically based pharmacokinetic (PBPK) models, lung, muscle, and liver were the primary deposition sites suggesting that bone is not a common storage site [123].

### Human milk

Arsenic concentrations in human milk are low compared to corresponding levels in blood or urine [6, 124–126]. For example, in a study of 10 Andean women with high levels of arsenic in drinking water (200 µg/l), median blood, urine, and human milk total arsenic concentrations were 9.6 µg As/l, 400 µg As/l, and 3.1 µg/kg As, respectively [124]. In a study of 88 women from the Faroe Islands, authors reported median human milk arsenic concentrations of 1.6 µg/kg that were not correlated with fish intake suggesting minimal transfer of organic arsenic from seafood to the milk [126].

## Research priorities and data gaps

Our review identified the following questions that represent knowledge/data gaps and research priorities.

- What is the appropriate interpretation of DMA?

As noted in Table 1, DMA can be directly consumed, metabolized from organic arsenic, or metabolized from methylation of iAs and MMA and is, therefore, not a reliable biomarker of iAs exposure in populations with low levels of exposure [23] or moderate seafood consumption [74]. Toxicokinetic studies in pregnancy with detailed dietary exposure and biomonitoring data may help address this issue by investigating arsenic excretion patterns in pregnant women with low levels of exposure [55, 127]. According to the US ATSDR Toxicological Profile [6], there are no identified PBPK studies of pregnant or lactating women. Studies of arsenic metabolism have been conducted in adult males [128] or rodents [129]. In the absence of these toxicokinetic data, researchers need to consider whether their research question is concerned with systemic DMA and all the varied sources it captures or whether they are interpreting DMA as a biomarker of iAs exposure. Caution is advised against the latter interpretation in low-exposed populations or those with moderate seafood consumption. As previously noted, analytical methods (statistical adjustment, restriction, or calibration) are advised to account for the potential influence of seafood consumption and resulting DMA exposure from arsenicals in seafood when using the sum of iAs, DMA and MMA as a biomarker of total iAs exposure [74, 75]. Previous investigations of the health effects of DMA have been based on urinary concentrations [106, 130]; however, the same challenge would apply to other matrices.

- How do iAs concentrations change throughout pregnancy and what are the advantages of measurement in each trimester?

**Fig. 2:**
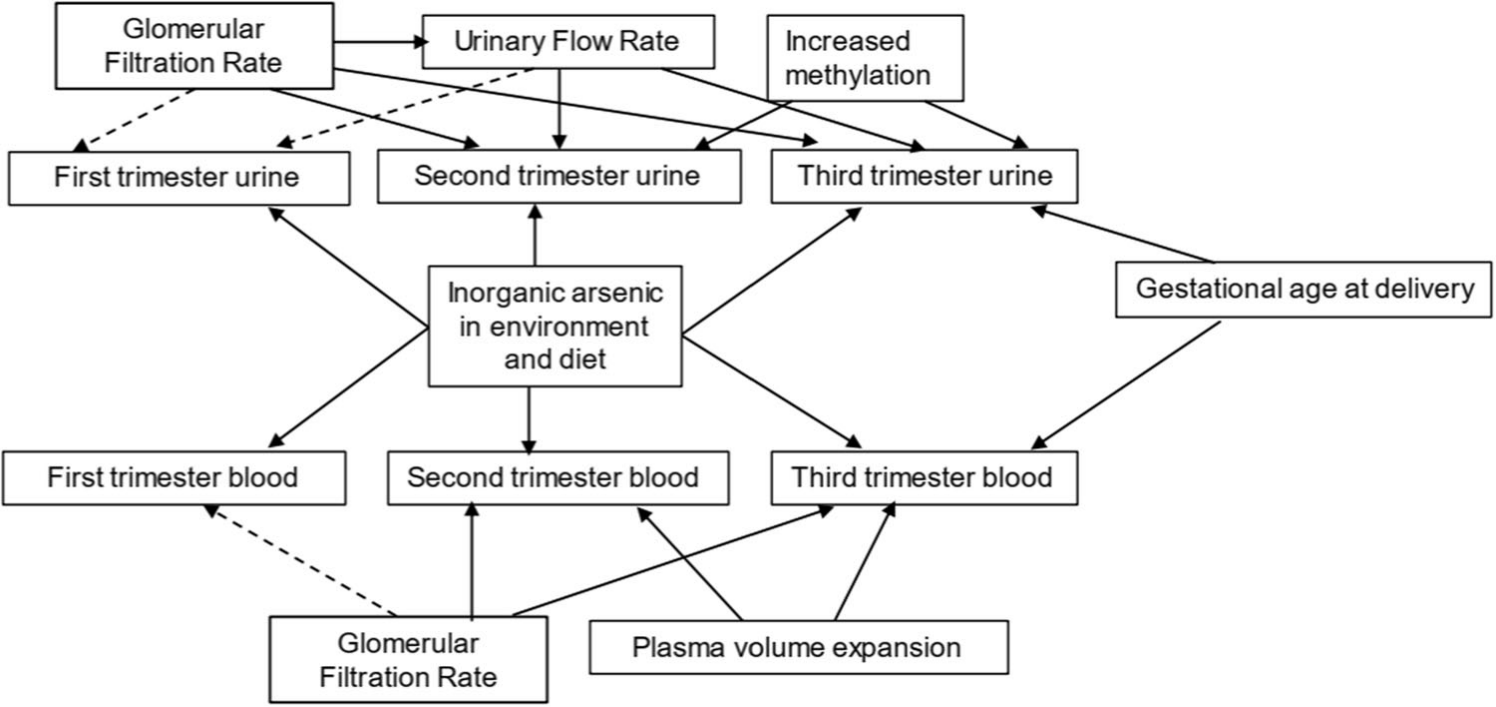
Conceptual model illustrating factors that influence biomonitoring data in blood and urine samples during each trimester. Urine samples may be influenced by glomerular filtration rate and urinary flow rate. The impact on first trimester concentrations is likely minimal in the first trimester compared to the second or third trimesters and is indicated by a dashed line. As pregnancy continues, methylation efficiency increases and may influence the speciation profile observed in the second and third trimester urinary concentrations. GFR may also influence blood concentrations in each trimester with the second and third trimester concentrations being most affected. Plasma volume expansion in the second and third trimester will also influence blood concentrations during these time points. Third trimester blood and urine samples may not be available if delivery occurs at any early gestational age.

Much of the research on iAs exposure throughout pregnancy has been done in highly exposed populations (i.e., arsenic drinking water concentrations ≥10 µg/l) and has been based on total arsenic urinary concentrations. Data are scarce regarding the potential change in blood or speciated urinary concentrations from early to late gestation. Serial measurements are important not only for determining if one measurement accurately reflects exposure throughout pregnancy but also for understanding the influence of the physiological changes of pregnancy on biomarker concentrations. As shown in Fig. 2, blood and urinary concentrations in each trimester may be influenced by physiological changes of pregnancy. First trimester urinary speciated arsenic concentrations reflect exposure patterns several days prior to sample collection and may not capture exposure profiles during the relevant physiological window. Similarly, first trimester blood samples will reflect exposure at the time of sample collection and speciation may not be feasible particularly in low-exposed populations. Collecting samples in the second and third trimester lengthens the window of estimated exposure levels but these samples may be more prone to resulting misclassification bias due to urinary flow rate or GFR, plasma volume expansion, and increasing methylation efficiency throughout pregnancy. Corrections for kidney function reduce, but may not completely eliminate, this source of misclassification bias [85]. Furthermore, third trimester samples may be censored due to early delivery and would, therefore, not be suitable for analysis of birth outcomes such as preterm birth. Serial measurements of iAs throughout pregnancy and in multiple matrices will help understand temporal stability, the influences of pregnancy-induced physiological changes on iAs biomarker concentrations and identify critical windows of exposure.

As noted previously, literature regarding the correlation of serial toenail or hair sample has been performed in highly exposed populations [47, 102] and replication of these findings in low-exposed populations is necessary to determine the suitability of using one postpartum sample to reflect average exposure throughout pregnancy.

- Why invest in development of methods to measure iAs in toenails and hair?

Toenails and hair are novel matrices that offer several potential advantages over the more well-established matrices such as blood and urine; however, the lack of standard reference material and processing protocols for these matrices impedes comparison of concentrations among studies and interpretation of results [45]. Adoption of standardized laboratory methods would enhance the contribution of data from these matrices to exposure assessment and etiological studies. Considering the ease of collection, storage, and shipment, toenails and hair could be collected remotely and allow researchers to overcome current and potential future pandemic-imposed restrictions on clinic based biospecimen collection. In addition, nails and hair will provide longer-term assessments of iAs exposure rather than the snapshots of excretion and recent exposures provided by urine and blood concentrations. Compared to urine, these matrices are also less subject to potential confounding due to seafood intake because the speciation profile is dominated by iAs rather than DMA. There is, therefore, considerable rationale for developing standardized laboratory methods for these novel matrices.

- Can iAs species be reliably measured in blood in low-exposed populations?

Several studies have measured iAs species in blood [91–93], yet these methods have not been applied in low-exposed populations. The current reliance on total As in blood is a barrier to understanding iAs toxicity and exposure levels. Development of speciation methods in low-exposed populations would be a considerable benefit to exposure assessment and epidemiological studies.

Although not a focus of the present review, we acknowledge that knowledge gaps exist regarding the bioavailability and health risks of organic arsenicals (e.g., arsenocholine, arsenobetaine) [22, 131, 132]. We also recognize that there are numerous other data gaps regarding the measurement and health risks of arsenic exposure. The role of nutritional factors (e.g., folate and selenium) [54, 57, 58, 133] and genotype (e.g., single nucleotide polymorphisms of enzymes involved in arsenic metabolism (arsenic methyltransferase (As3MT), DNA-methyl-transferase)) [134, 135] on speciation profiles during pregnancy both warrant further attention but were beyond the focus of this review.

## Conclusions

Due to the complexity of arsenic metabolism and the physiological changes of pregnancy, exposure assessment during pregnancy is challenging. Continued investigation into arsenic toxicokinetics during pregnancy and development of sensitive laboratory methods, as well as careful interpretation of biomonitoring data are necessary to advance scientific understanding of exposure levels throughout pregnancy and the etiological role of low-level arsenic exposure in maternal-child health outcomes. Advancing scientific understanding of arsenic body burden in pregnancy is necessary because diet continues to be a source of exposure and because it is not known whether a no effects level exists for arsenic in pregnancy. We encourage epidemiologists to account for the unique physiology of pregnancy when interpreting biomonitoring data and encourage analytical chemists to further develop and standardize laboratory methods for measurement of speciated arsenic in novel matrices.
